# Wearable Sensors for Assessing the Role of Olfactory Training on the Autonomic Response to Olfactory Stimulation

**DOI:** 10.3390/s21030770

**Published:** 2021-01-24

**Authors:** Alessandro Tonacci, Lucia Billeci, Irene Di Mambro, Roberto Marangoni, Chiara Sanmartin, Francesca Venturi

**Affiliations:** 1 Institute of Clinical Physiology, National Research Council of Italy (IFC-CNR), 56124 Pisa, Italy; atonacci@ifc.cnr.it; 2 School of Engineering, University of Pisa, 56122 Pisa, Italy; i.dimambro@studenti.unipi.it; 3 Department of Biology, University of Pisa, 56127 Pisa, Italy; roberto.marangoni@unipi.it; 4 Institute of Biophysics, National Resarch Council of Italy (IBF-CNR), Via Moruzzi 1, 56124 Pisa, Italy; 5 Department of Agriculture, Food and Environment, University of Pisa, 56124 Pisa, Italy; chiara.sanmartin@unipi.it (C.S.); francesca.venturi@unipi.it (F.V.); 6 NexFood Srl, 57121 Livorno, Italy

**Keywords:** autonomic nervous system, electrocardiogram, galvanic skin response, olfactory training, psychophysics, smell, wearable sensors, wine sensory analysis

## Abstract

Wearable sensors are nowadays largely employed to assess physiological signals derived from the human body without representing a burden in terms of obtrusiveness. One of the most intriguing fields of application for such systems include the assessment of physiological responses to sensory stimuli. In this specific regard, it is not yet known which are the main psychophysiological drivers of olfactory-related pleasantness, as the current literature has demonstrated the relationship between odor familiarity and odor valence, but has not clarified the consequentiality between the two domains. Here, we enrolled a group of university students to whom olfactory training lasting 3 months was administered. Thanks to the analysis of electrocardiogram (ECG) and galvanic skin response (GSR) signals at the beginning and at the end of the training period, we observed different autonomic responses, with higher parasympathetically-mediated response at the end of the period with respect to the first evaluation. This possibly suggests that an increased familiarity to the proposed stimuli would lead to a higher tendency towards relaxation. Such results could suggest potential applications to other domains, including personalized treatments based on odors and foods in neuropsychiatric and eating disorders.

## 1. Introduction

As recently reviewed by Kryklywy and co-workers [1], from an evolutionary point of view, representations of valence-labeled sensation in emotion-processing regions are not an ancillary feature developed to inform centralized affect representation. Rather, they reflect the ancestral role of these structures; they are relics of a time when the experiences of sensory information and emotional-motivational states were one and the same.

While the whole mechanism still seems far from being fully understood, interactions between cortical and thalamic regions appear to play a key role in cognitive functions, with several lines of research now suggesting that a fundamental aspect of thalamic functioning is that it fuses perceptual, emotional and cognitive information into one single meaningful experience [2].

More than any other sensory modality, olfaction is like emotion in attributing positive (appetitive) or negative (aversive) valence to the environment, and the close anatomic relations between the systems deployed for olfaction and for emotion [3] account for the important links found between these two functions [4,5,6,7].

In this context, in the clinical field the sense of smell is mainly related to neurodegenerative processes, where olfactory impairments are reported, somewhat linked to the disease progression [8].

Notably, abnormal olfactory processing is also seen in other neurodevelopmental [9] and neuropsychiatric disorders, including those where an impaired attitude towards feeding represents one of the hallmarks of the condition [10].

Among them, anorexia (AN) and bulimia nervosa (BN) experience, especially in more severe cases, a peculiar olfactory processing, made up of distorted sensitivity and reactivity to sensory stimulation, possibly related to the food aversion typical of those individuals [10,11,12].

Recently, a significant literature has been focused on the positive effects of olfactory training [13]. Indeed, the unprecedented worldwide pandemic represented by COVID-19 has put anosmia and dysgeusia at the forefront of clinical investigation, the loss of smell and taste being some of the most prevalent side effects of the infection, often occurring also in asymptomatic or pauci-symptomatic subjects, possibly representing a biomarker of infection occurrence and, somewhat, severity [14,15]. In this specific domain, Liu et al. [16] demonstrated the capability of the sense of smell to regenerate, in turn contributing to an overall improvement of the quality of life in individuals already challenged by the diverse physically-, psychologically- and socially-disrupting consequences of the COVID-19 infection [17,18,19].

Until now, olfactory training was seen to be effective in individuals where a significant related sensory impairment is present; however, it was never analyzed in terms of enhancement of the sensory function in individuals where the olfactory pathway is already optimal, nor in relation to psycho-physiological changes eventually occurring in the human body following the modification of odor familiarity and/or valence, two dimensions of the olfactory stimulus often neglected but worth investigation.

Previous studies conducted on healthy subjects have hypothesized the existence of a significant correlation between odor familiarity and pleasantness, intended as the positive attitude experienced by an individual towards an odorous compound [20,21,22]. However, those works have not clarified exhaustively and objectively the consequentiality between those two domains of the olfactory processing. This represents a significant gap in the current literature, since knowing how those two domains interact to each other can be critical to thinking about possible therapeutic strategies based on sensory, particularly olfactory, stimulation, addressed to several disorders where an abnormal attitude towards feeding represents one of the main clinical features.

To objectively and quantitatively assess the physiological response to olfactory stimulation, several strategies have been adopted throughout the years, with a number of either invasive or unobtrusive techniques applied, each displaying significant drawbacks in terms of applicability, acceptability and informativity [23,24,25]. A reasonable solution, merging acceptability, low cost and reliability and providing useful information about the physiological reactions to odorous stimuli is represented by the assessment of biomedical signals triggered by the activity of the autonomic nervous system (ANS), including electrocardiogram (ECG) and galvanic skin response (GSR), already studied in relationship with the olfactory assessment [26,27]. Such signals can be acquired in a completely non-invasive manner using wearable sensors, as demonstrated in several literature works published to date (e.g., [26,27]).

Therefore, the aim of the present article is to investigate the relationship between odor familiarity and pleasantness, the latter studied not using basic questionnaires, possibly associated with conscious responses and subsequent biases, but evaluating the activation of the ANS in a cohort of healthy individuals. This first approach is mandatory to understand, net of any possible clinical condition, the relationship between the two odor-related domains paving the way, in case of significant correlations, for more tailored investigations on specific clinical disorders.

## 2. Materials and Methods

### 2.1. Selection and Training of Panelists

For this study, 25 students (9 females and 16 males; ranging from 21 to 29 years old) enrolled in the bachelor degree in “Oenology and Viticulture” of the University of Pisa (Italy) were initially recruited based on their motivation and willingness.

Selection and training of panelists was performed according to the University of Pisa, Department of Agriculture, Food and Environment (DAFE) internal procedure, which is based on a normalized technical procedure reported in literature with some modifications [21]. The trained panelists are then included in the official panel of the DAFE. They have to repeat and pass re-qualification tests once a year, considering their efficacy as tasting judge, in terms of their own repeatability, discrimination ability and compliance. Re-qualification tests, in addition to providing information about panelists’ suitability, help to keep the panelists alert, avoiding relaxation and undervaluation of training.

A multi-step training period was therefore arranged in order to select a group of students characterized by the necessary motivation during the whole activity (attendance at more than 75% of training sessions) together with the minimum sensory skills required for wine tasting and description (including visual, aroma and taste attributes).

The training of the 25 students was arranged as follows over a period of three months:

(i) Step 1 (15 h): Theoretical introduction to the principles of human physiology of sight, smell and taste.
(ii) Step 2 (20 h): Arrangement of preliminary training tests, mainly based on the utilization of model standard solutions, to collect information about the tasting capacity of each panelist (i.e., sensory acuity (detection thresholds); odor and flavor memory; term use and recall; scoring consistency).
(iii) Step 3 (30 h): As discrimination is probably based as much on odor memory (that accumulates with experience) as on sensory acuity, ten wine tasting sessions were carried out in the morning, in a well-ventilated quiet room and in a relaxed atmosphere. During each of the ten tasting sessions, the panelists evaluated three different commercial wines (globally thirty different wines were assessed including white, rosé and red wines). The assessors used a sensorial sheet, specifically developed for this purpose, consisting of a non-structured, parametric, descriptive wine scoring chart [28]. Before starting the sensory evaluations, panelists were provided with the synthetic definitions of each descriptors proposed in the sensorial sheet. Furthermore, the panelists were also asked to freely describe the specific olfactory expression of each tasted wine to familiarize themselves with the main descriptors generally utilized for wine’s sensory analysis [29].

**Figure 1:**
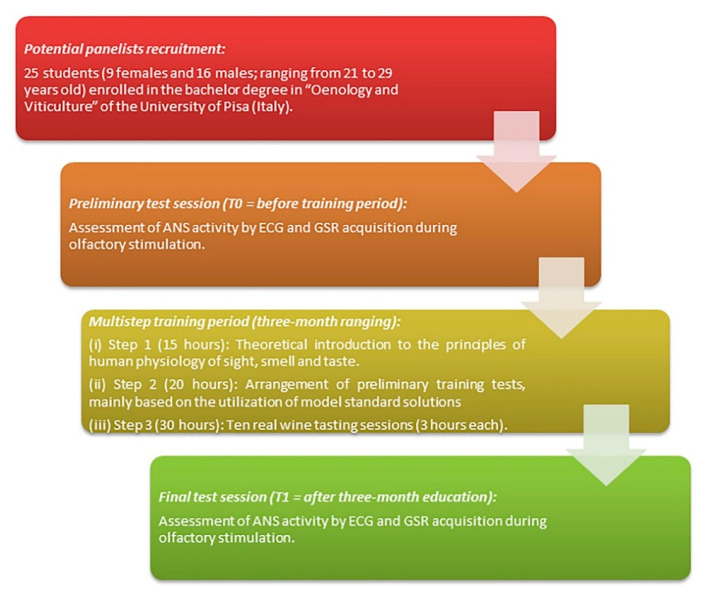
Experimental design.

The overall experimental design, including the sensory assessment, is displayed in Figure 1.

### 2.2. Model Solutions Used for the Olfactory Stimulation

To reproduce, as much as possible, the main olfactory sensations that are mostly utilized for the description of wine’s olfactory behavior, some model solutions were prepared in an affordable and easily reproducible way by utilizing raw material widely available at the supermarket (i.e., commercial fruit juices; fresh fruits and vegetables; distilled flower water). Furthermore, as threshold values are significantly influenced by the solvent (i.e., water vs. ethanol) model solutions were prepared in a neutral white wine base.

As during tasting experience both synergistic and suppressive influences among different wine’s aromatic compounds must be always taken into account, our approach allowed us to create a more real tasting experience than what could have been obtained by utilizing model solutions produced starting from chemical pure standards diluted in artificial model wine.

In Table 1, the odorous solutions used for the olfactory stimulation are reported.

Among them, a subset of 10 odorants were employed for the testing sessions. This choice was performed for two main reasons: (i) to reduce the duration of testing, therefore avoiding problems related to the conditioning of physiological signals by the fatigue or annoyance eventually felt by the panelists, and (ii) to keep significant information about the physiological response to a “representative” subset of odors, with the odorants selected belonging to different families (e.g., fruity, floral, etc.).

To such extent, the ten odorants finally selected for testing are displayed in Table 2.

### 2.3. Testing Procedure

Two test sessions were carried out for the present study, respectively, namely before the training period (T0), and after the three-month education (T1).

In both sessions, an assessment of ANS activity was performed using wearable sensors, as later described, within three different sessions:

(i) Baseline (3′ duration): At baseline, the subjects were comfortably sitting on a chair and asked to relax.
(ii) Task (6′ 40” duration): At task, 10 model solutions were administered to the panelists for odors detection for 10” each, with an inter-stimulus interval of 30” to allow cleaning the nasal cavity from the previous odor [30] as well to let the GSR signal return to the baseline condition. The subjects were asked to report the identifier for each of the odor presented on a paper sheet.

### 2.4. ANS Assessment

The assessment of the ANS activity was performed studying physiological signals acquired by wearable sensors.

Notably, two signals of interest to this extent included (i) the ECG, one of the most important biomedical signals as it relies on the electrical activity of the heart, and (ii) the GSR, related to the electrical activity of the skin caused by the activation of the sweat glands.

Both those signals are normally correlated with the activation of the ANS, thus representing a useful, non-invasive means to assess its functioning, as demonstrated in [26,27].

#### 2.4.1. ECG Acquisition and Processing

The acquisition of the ECG signal was performed using a commercial wearable, Bluetooth-equipped sensor, named Shimmer ECG (Shimmer Sensing, Dublin, Republic of Ireland), attached to a commercial fitness-like chest strap (Polar Electro Oy, Kempele, Finland). In order to comply with the international guidelines for the estimation of the Heart Rate (HR) and its variability (heart rate variability (HRV)) [31], the ECG signal was acquired at 500 Hz, not requiring particular controls about the battery duration given the structured experimental setting.

The ECG signal was analyzed using a dedicated routine implemented in MATLAB (The MathWorks, Inc., Natick, MA, USA) [32] and optimized for the present work.

**Figure 2:**
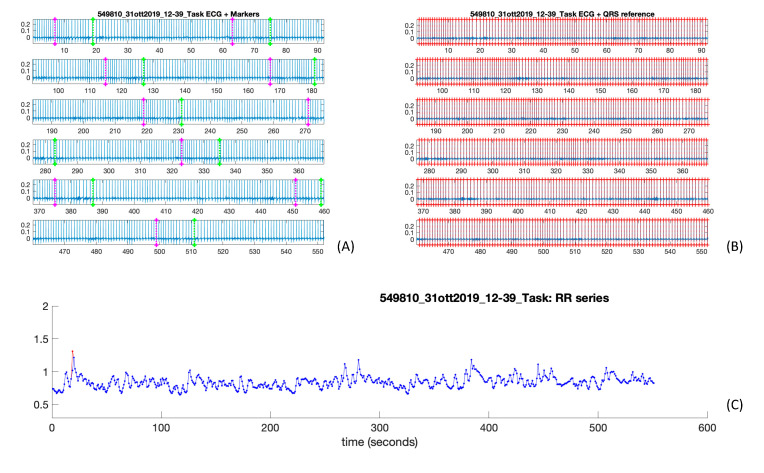
ECG signal processing for a sample data: (**A**) original ECG signal with the markers indicating the start (purple) and the end (green) of each odor stimulation;(**B**) the same signal after the application of the QRS complexes detector (QRS markers indicated in red); (**C**) the RR series obtained from the ECG signal before (red) and after (blue) correction.

ECG signals were pre-processed for artifact removal, QRS complexes were detected and then the RR series were reconstructed and corrected (for an example, see Figure 2). The correction was applied to remove correction of non-sinusoidal beats in order to obtain an RR series that only contains variations due to the sinus node and thus reflects the activity of the ANS [32]. From the corrected RR series, a number of significant features were extracted, notably:

- Time-domain features:
  - Heart rate (HR): number of heart beats per unit of time. Measured in beats per minute (bpm), it is usually associated with the sympathetic branch of the ANS [33];
  - Standard deviation of the normal R–R intervals (SDNN): measured in ms, it is an estimate of the HRV influenced by both the sympathetic and para-sympathetic branches of the ANS [33];
  - Root mean square of the successive differences (RMSSD): measured in ms, it represents the root mean square of the differences between neighboring R–R intervals. It is an estimate of the parasympathetic activity of the ANS [33];
  - Number of normal R–R intervals differing for more than 50 ms (NN50): it estimates the number (or the percentage) of the normal R–R intervals differing for more than 50 ms from each other. Under resting state short-term recordings, it refers to the parasympathetic activity of the ANS [33];
  - Variance of the R–R intervals (VAR): it refers to the variability of the R–R intervals;
  - SD1: standard deviation of the projection of the Poincaré plot on the perpendicular line to the identity. It estimates the short-term HRV;
  - SD2: standard deviation of the projection of the Poincaré plot on the parallel line to the identity. It estimates the long-term HRV;
  - Cardiac sympathetic index (CSI): obtained by the Poincaré plot and calculated as SD2/SD1, it is employed as a reliable indicator of the sympathetic activity of the ANS [34];
  - Cardiac vagal index (CVI): obtained by the Poincaré plot and calculated as log10 (SD1 × SD2), it is employed as a reliable indicator of the parasympathetic activity of the ANS [34].
- Frequency–domain features:
  - Low frequency (LF): power spectral density of the ECG signal at low frequencies (0.04–0.15 Hz), it is employed as an estimator of the sympathetic activity of the ANS [33];
  - High frequency (HF): power spectral density of the ECG signal at high frequencies (0.15–0.4 Hz), it is employed as an estimator of the sympathetic and parasympathetic activity of the ANS [33];
  - Low-to-high frequency components ratio (LF/HF): it indicates the overall balance between low and high frequency components of the ECG signal. A ratio exceeding 1 suggests a sympathetic dominance, whereas for values below 1, the parasympathetic nervous system appears to be prevalently activated [33]. It should be stated that the reliability of the LF/HF ratio in quantifying the overall sympathetic/parasympathetic balance is often questioned by several works in the scientific literature, as it is judged less accurately and is more affected by artifacts than what occur with time-domain features [35].

#### 2.4.2. GSR Acquisition and Processing

The acquisition of the GSR signal was conducted with a commercial wearable sensor, Shimmer3GSR (Shimmer Sensing, Dublin, Republic of Ireland), communicating via Bluetooth to the manufacturer user interface. Here, the sampling frequency was kept at 51.2 Hz, which was one of the higher with respect to the available choices allowed by the sensor firmware and in compliance with previously published protocols [36]. The GSR sensor captured the corresponding signal, being attached to two adjacent fingers of the subject’s non-dominant hand at the phalanx level with the support of two comfortable soft rings in turn worn by the individual studied.

**Figure 3:**
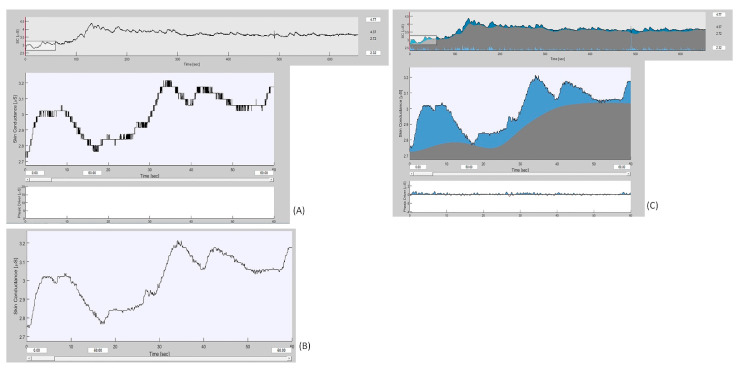
Galvanic skin response (GSR) signal processing: (**A**) the Ledalab interface displaying the raw signal (upper plot) and a 60-s portion of it (medium plot); (**B**) the same 60-s portion after the application of the first order Butterworth low-pass filter at 5 Hz; (**C**) the signal filtered (upper plot), the 60-s portion (medium plot) and the phasic component extraction after applying a continuous decomposition analysis (lower plot).

Concerning the processing, the GSR signal was analyzed using Ledalab, a MATLAB-based tool devoted to the processing of this specific biomedical signal [37]. The GSR signal was filtered at first with a first order Butterworth low-pass filter at 5 Hz to remove high frequency noise, and then continuous decomposition analysis was applied for the extraction of both tonic and phasic activities (see Figure 3 for an example). As such, the following features were extracted:

- Global GSR signal: composed of the sum of the tonic and phasic components of the signal;
- Tonic GSR component: mainly refers to slow changes of the electrical skin signal, dominant at rest and during relaxing activities not including specific stimuli;
- Phasic GSR component: extracted to study the response to the sensory (olfactory) stimulation, as it refers to quick responses to specific stimuli. It is often termed skin conductance response (SCR).

### 2.5. Statistical Analysis

Data normality was assessed using the Shapiro–Wilk test for each of the parameters studied [38]. In case of parameters displaying a normal distribution, Student’s t-test was applied to compare couples (e.g., scores at baseline vs. task, or at T0 vs. T1), whereas with data deviating from normality, the Wilcoxon signed-rank test was applied.

For all the analyses conducted, statistical significance was set at *p* < 0.05.

## 3. Results

### 3.1. ECG Signal

In the ECG signal, the features extracted and described above were analyzed in terms of the comparison between T0 and T1 of the baseline, as well as within each testing session (e.g., at T0 and at T1) in terms of the comparison between baseline and task. Due to the nature of the HRV, it is quite infrequent (and useless) to calculate HRV features within short time windows like the ones dealing with the odorous stimulation (e.g., 10 s); therefore at both T0 and T1, the task phase was further divided into task ON and task OFF, being, respectively, the portion of the task phase where the odorous stimulation took place and the inter-stimulus portion of the task phase.

The results obtained are displayed in Table 3.

### 3.2. GSR Signal

As for the GSR signal, comparisons between baseline at T0 and at T1, as well between baseline at T0 and task at T0 (and baseline at T1 and task at T1) were conducted evaluating differences arising in terms of global and tonic GSR. The responses to single odorants at T0 with respect to T1 were compared by means of the phasic component of the GSR. This analysis, slightly different with respect to those carried out with the ECG signal, was possible thanks to the fact that the GSR analysis, particularly concerning the tonic phase evaluation, can be performed also on very short time windows like the ones represented by the 10 s of olfactory stimulation foreseen in the present protocol.

The results obtained are displayed in Table 4.

On the other hand, no significant differences between the GSR phasic signals were seen for any of the odorants administered at T1 with respect to T0.

## 4. Discussion and Conclusions

The analysis of such results should be performed taking into account the physiological meanings of both ECG and GSR signals and their ability to map the activity of the ANS.

Notably, the various features extracted from the ECG signal can reliably detect changes occurring at both the sympathetic and parasympathetic branches of the ANS (see [33] for a review), whereas the GSR signal is capable of monitoring the sympathetic arousal [39], albeit often reported to be less sensitive to subtle modifications of the ANS activity with respect to the ECG-related features [40,41,42].

In fact, more specifically, it is well known that different components of the ANS activated by emotional reactions can be detected by both signals. Concerning the ECG, the heart rhythm and its variability are modulated by the activity of both sympathetic and parasympathetic nerves, whereas the GSR only reflects the activation of the sympathetic branch of the ANS, probably representing the elective method for assessing the emotional arousal [43]. Several works have demonstrated the higher performance of the ECG signal with respect to the GSR in representing a useful indicator of emotions (see [44]) for some related description), but the use of both signals is basically justified by their informative value.

As such, it is reasonable that slight, sometimes pleasant sensory stimulations, as represented by olfactory stimuli, could drive quite subtle changes in the ANS activity, therefore requiring a comprehensive analysis of both ECG and GSR signals to be appreciated, at least in individuals with preserved sensory ability.

Overall, the results obtained in the present work suggest that the engagement in an emotionally-demanding task like sniffing an odorant might drive the ANS towards a higher sympathetic activation and somewhat vagal withdrawal in cognitively- and sensory-intact individuals. Indeed, both the ECG and GSR features are consistent about this point, especially during the first session (T0), conducted prior to the olfactory training. Such a result appears consistent with previous literature works, proving once more this existing, albeit subtle association [26,45].

Interestingly, ECG features were also able to retrieve some differences between the “sub-phases” of the task, displaying higher vagal activation while sniffing and higher sympathetic activity during inter-stimulus phases, where individuals are engaged in a more cognitively demanding task, again consistently with the literature [26].

The effect noticed comparing results at the two testing sessions also suggests a slightly different behavior at T1 with respect to T0. In fact, the higher vagal withdrawal highlighted by RMSSD at T1 during inter-stimuli with respect to the olfactory stimulation sub-phase, and the absence of any sympathetically-driven arousal detectable by the tonic GSR signal suggest that the more odors become familiar to the panelists (as occurring after the training period, at T1, in our protocol), the more they show a tendency to provoke higher vagal responses in the individuals evaluated.

Such results lead to the consideration about a clear link between familiarity and pleasantness [46], the latter being studied through the “implicit” assessment of the ANS [47].

Generally, sensory channels, at least concerning unpleasant triggers, display an adaptation for repeated stimulations, with lower autonomic reactivity as much as the stimulus becomes familiar. However, this was demonstrated only limited to unpleasant stimuli and excluding the olfactory channel [48], this domain remaining yet poorly explored in the literature.

The results obtained in the present article go beyond such retrievals and makes sense also from an evolutionary perspective. Indeed, the main survival role of the olfactory system is to identify potential environmental hazards and to adopt the optimal approach to the surrounding universe [49], including that with respect to feeding, activating an approach/avoidance behavior, which is critical to ensure the individual’s survival [50,51]. As such, odors are highly capable of eliciting affective responses and emotions, thanks to the direct connections between primary olfactory areas and the areas of the limbic systems, without a thalamic relay [52], making olfaction a particularly useful, non-invasive mean to somewhat access some specific information processed by specific areas of the brain.

Proving, using an implicit methodology and thus relying on the study of the autonomic changes brought by odors, that olfactory training, beyond enhancing familiarity, also leads to different autonomic reactivity, particularly on domains concerned with odor pleasantness, can be of critical importance from a clinical perspective. Indeed, although this work has demonstrated the association only on healthy individuals, and in particularly university students, which are probably interested and more favorable to the present approach, it could form the basis for future investigations on clinically relevant cohorts.

Administering a similar protocol, for example, to subjects characterized by disturbed sensoriality, in turn leading to abnormal behavior towards feeding, such as extreme food aversion, binge eating or overfeeding, would help in understanding eventual autonomic abnormalities in such specific cohorts and eventually in hypothesizing a treatment based on repeated olfactory stimuli tailored on the specific disease phenotype and, in some instances, on an individual-basis. If proven to be beneficial, this would represent an undoubted advancement in the current clinical practice that the development of Information and Communication Technology would enable.

## Figures and Tables

**Table 1:** Odorous solutions used for the stimulation.

Sample Code	Descriptor	Formulation
1	Apricot	80 mL of commercial juice (brand: Skipper Zuegg, 40% min. fruit pulp) + 100 mL of white table wine *
2	Berries	80 mL of commercial juice (brand: Skipper Zuegg, 30% min. fruit pulp) + 100 mL of white table wine *
3	Blueberry	80 mL of commercial juice (brand: Skipper Zuegg, 40% min. fruit pulp) + 100 mL of white table wine *
4	Raspberry	80 mL of commercial juice (brand: Esselunga Bio, 45% min. fruit pulp) + 100 mL of white table wine *
5	Grapefruit	80 mL of commercial juice (brand: Esselunga Bio, 45% min. fruit pulp) + 100 mL of white table wine *
6	Orange	80 mL of commercial juice (brand: Skipper Zuegg, 65% min. fruit pulp) + 100 mL of white table wine *
7	Pineapple	80 mL of commercial juice (brand: Skipper Zuegg, 55% min. fruit pulp) + 100 mL of white table wine *
8	Figs	10 g dried figs without dilution
9	Walnut	Maceration ** of 25 g walnut kernels in 100 mL of white table wine *
10	Asparagus	60 mL of cooking water + 100 mL of white table wine *
11	Peach	80 mL of commercial juice (brand: Skipper Zuegg, 65% min. fruit pulp) + 100 mL of white table wine *
12	Apple	80 mL of commercial juice (brand: Skipper Zuegg, 85% min. fruit pulp) + 100 mL of white table wine *
13	Pear	80 mL of commercial juice (brand: Skipper Zuegg, 65% min. fruit pulp) + 100 mL of white table wine *
14	Green pepper	Maceration ** of 20 g of fresh green pepper in 100 mL of white table wine *
15	Banana	Maceration ** of 20 g of banana pulp in 100 mL of white table wine *
16	Mango	Maceration ** of 5 g of dried mango in 100 mL of white table wine *
17	Plum	Maceration ** of 5 g of dried plums in 100 mL of white table wine *
18	Lemon	50 mL of commercial juice (brand: Eurofood, 100% lemon juice) + 100 mL of white table wine *
19	Mix of exotic fruit	80 mL of commercial juice (brand: Skipper Zuegg, 55% min. fruit pulp) + 100 mL of white table wine *
20	Zagara	8 mL of distilled orange blossom water + 100 mL of white table wine *
21	Rose	4 mL of distilled rose water + 100 mL of white table wine *

* Brand: Tavernello. ** Maceration conditions: storage for 24 h in the dark at room temperature (20 ± 1 °C).

**Table 2:** Odorants selected for the testing sessions.

Sample Code	Descriptor
4	Raspberry
5	Grapefruit
6	Orange
7	Pineapple
8	Figs
10	Asparagus
11	Peach
14	Green pepper
16	Mango
21	Rose

**Table 3:** Main results concerning the ECG features throughout the protocol phases (B: baseline, T OFF: task inter-stimulus, T ON: task with olfactory stimulation; *: *p* < 0.05; **: *p* < 0.01; n.s.: not significant).

Feature	B T0	B T1	T ON T0	T ON T1	T OFF T0	T OFF T1	B T0 vs. B T1	B T0 vs. T ON T0	B T1 vs. T ON T1	T ON T0 vs. T OFF T0	T ON T1 vs. T OFF T1
HR (bpm)	71.9 ± 10.6	67.7 ± 9.4	78.8 ± 12.1	75.1 ± 9.0	78.3 ± 9.6	74.0 ± 7.1	n.s.	0.005 **	<0.001 **	n.s.	n.s.
RMSSD (ms)	0.063 ± 0.031	0.071 ± 0.031	0.083 ± 0.055	0.067 ± 0.031	0.063 ± 0.045	0.050 ± 0.015	n.s.	n.s.	n.s.	n.s.	0.018*
NN50	18.9 ± 12.8	47.8 ± 34.4	4.4 ± 2.1	4.5 ± 1.8	12.1 ± 7.1	9.1 ± 4.4	0.036 *	0.002 **	0.002 **	<0.001 **	<0.001 **
SD1	0.044 ± 0.022	0.050 ± 0.022	0.058 ± 0.039	0.047 ± 0.022	0.044 ± 0.032	0.035 ± 0.011	n.s.	n.s.	n.s.	n.s.	0.018 *
CSI	2.238 ± 0.770	2.100 ± 0.546	2.224 ± 0.939	2.346 ± 0.512	2.825 ± 0.919	2.783 ± 0.451	n.s.	n.s.	n.s.	0.020*	0.036 *
CVI	−2.490 ± 0.362	−2.361 ± 0.309	−1.510 ± 0.521	−2.418 ± 0.351	−2.474 ± 0.444	−2.545 ± 0.260	n.s.	n.s.	n.s.	n.s.	0.007 **
LF (ms^2^)^2^/Hz	0.264 ± 0.167	0.233 ± 0.048	0.119 ± 0.031	0.098 ± 0.021	0.240 ± 0.049	0.237 ± 0.046	n.s.	0.004 **	<0.001 **	0.017 *	<0.001 **
HF (ms^2^)^2^/Hz	0.255 ± 0.450	0.802 ± 0.569	0.255 ± 0.156	0.288 ± 0.195	0.329 ± 0.318	0.351 ± 0.216	n.s.	0.010*	0.001 **	n.s.	n.s.
LF/HF	2.364 ± 3.620	0.535 ± 0.458	1.227 ± 1.479	0.098 ± 0.021	1.969 ± 1.769	1.177 ± 0.687	n.s.	n.s.	n.s.	0.013*	0.016 *

**Table 4:** Main results concerning the GSR signal analysis (B: baseline, T: task; *: *p* < 0.05; **: *p* < 0.01; n.s.: not significant).

Feature	B T0	B T1	T T0	T T1	B T0 vs. B T1	B T0 vs. T T0	B T1 vs. T T1
GSR global (µS)	2.13 ± 2.33	3.75 ± 4.90	3.09 ± 3.16	4.73 ± 6.88	n.s.	0.015 *	n.s.
GSR tonic (µS)	1.93 ± 2.14	3.56 ± 4.64	2.84 ± 2.73	4.53 ± 6.58	n.s.	0.009 **	n.s.

## Data Availability

The data presented in this study are available on request from the corresponding author. The data are not publicly available due to ethical reasons.
